# Providing IoT Services in Smart Cities through Dynamic Augmented Reality Markers

**DOI:** 10.3390/s150716083

**Published:** 2015-07-03

**Authors:** David Chaves-Diéguez, Alexandre Pellitero-Rivero, Daniel García-Coego, Francisco Javier González-Castaño, Pedro Salvador Rodríguez-Hernández, Óscar Piñeiro-Gómez, Felipe Gil-Castiñeira, Enrique Costa-Montenegro

**Affiliations:** 1 AtlantTIC, Universidade de Vigo, Rúa Maxwell S/N, 36310 Vigo, Spain; E-Mails: apellitero@gradiant.org (A.P.-R.); javier@det.uvigo.es (F.J.G.-C.); pedro@det.uvigo.es (P.S.R.-H.); kike@det.uvigo.es (E.C.-M.); 2 Galician Research and Development Center in Advanced Telecommunications (GRADIANT), Edif. CITEXVI local 14, Campus Universitario de Vigo, 36310 Vigo, Spain; E-Mails: dgarcia@gradiant.org (D.G.-C.); opineiro@gradiant.org (O.P.-G.); xil@det.uvigo.es (F.G.-C.)

**Keywords:** Internet of Things, sensors, augmented reality, smart city, visible light communication

## Abstract

Smart cities are expected to improve the quality of life of citizens by relying on new paradigms, such as the Internet of Things (IoT) and its capacity to manage and interconnect thousands of sensors and actuators scattered across the city. At the same time, mobile devices widely assist professional and personal everyday activities. A very good example of the potential of these devices for smart cities is their powerful support for intuitive service interfaces (such as those based on augmented reality (AR)) for non-expert users. In our work, we consider a scenario that combines IoT and AR within a smart city maintenance service to improve the accessibility of sensor and actuator devices in the field, where responsiveness is crucial. In it, depending on the location and needs of each service, data and commands will be transported by an urban communications network or consulted on the spot. Direct AR interaction with urban objects has already been described; it usually relies on 2D visual codes to deliver object identifiers (IDs) to the rendering device to identify object resources. These IDs allow information about the objects to be retrieved from a remote server. In this work, we present a novel solution that replaces static AR markers with dynamic markers based on LED communication, which can be decoded through cameras embedded in smartphones. These dynamic markers can directly deliver sensor information to the rendering device, on top of the object ID, without further network interaction.

## 1. Introduction

Smart cities are complex environments where several areas of innovation meet in order to substantially improve socioeconomic development and quality of life [1]. Economic innovations, technological tools that encourage people to participate in governance processes and Internet-enabled city infrastructure services and utilities form a thriving research field.

Although a widely-accepted definition of the concept of a smart city does not yet exist, there is a general agreement that one of the main goals of smart cities should be to improve the quality and efficiency of city services, while reducing operational costs and making better use of public resources [2].

In order to achieve these goals, vast networks of interconnected sensors are being deployed. Just a few examples are pollution detectors, water quality meters, noise indicators and power grid controllers. Actuators, such as water control valves or electrical switches, also need to be installed in order to both monitor and control public resources. The practical interconnection of such a large set of devices to fulfill specific optimization goals requires technological advances beyond the theoretical results for classic wireless sensor network (WSN) architectures. The increasing need for scalability and distributed control has led to the concept of the Internet of Things (IoT).

The IoT paradigm, which transforms any object or appliance into part of a connected network, thus dissolving the barriers between the physical and virtual worlds, has become the cornerstone of many services in a smart city, including more efficient maintenance tasks. With the support of distributed sensor and actuator networks, it provides staff in the field with information as needed. This has been demonstrated in city-wide testbeds, such as Smart Santander [3], or the smart cities of Padova [2], Nice [4] and Oulu [5].

As stated in essential works on the topic [6], one of the main benefits of a world where everything is connected is that technology forms an invisible part of people's lives. This is where augmented reality (AR) interfaces can make a difference compared with more traditional approaches.

Nowadays, city service maintenance agents often have difficulties identifying and fixing problems in city infrastructures, as there is little technology available to facilitate their work. The system described in this paper aims to facilitate this work through AR technology. We assume a scenario in which a sensor and actuator network is available throughout the city infrastructure.

Traditional AR interfaces rely on static markers to determine the position and identifier (ID) of the object to which virtual information is linked. However, in this work, we propose a novel mechanism based on visible light communication (VLC) that not only provides the same identification and positioning information as AR markers, but also allows the transmission of small amounts of arbitrary data as a technological enabler to create more efficient services combining the IoT and AR paradigms. A VLC-based solution allows one to overcome some of the limitations of other near-range communication technologies. VLC has a higher range than RFID or NFC, limited to tens of centimeters at most. Therefore, the terminal camera may capture a wider scene while receiving data. This, combined with the high directionality of VLC, enables the implementation of smartphone AR interfaces. Besides, the proposed solution is also generalistic, since all smartphones include a camera, whereas RFID tags require too specific reader technologies (note that NFC technology, howbeit included in many commercial smartphone models, is not always available). Higher range radio beacons based on Bluetooth or WiFi are lowly directional, so they do not support AR rendering well. In addition, radio waves often propagate through walls, while VLC transmissions are always confined within the room where the beacon is installed.

The rest of this paper is organized as follows: Section 2 briefly reviews previous related work in the fields of IoT and AR interfaces applied to smart cities and also addresses basic usage of VLC with commercial image capture devices. Section 3 presents the main contribution of this article, the design of a dynamic AR anchor to link IoT infrastructure and AR interfaces via VLC communications. Section 4 describes the proposed architecture that takes advantage of these technologies to improve maintenance efficiency in smart cities. Section 5 describes the laboratory testbed that emulates the smart city scenario to validate the proposal and evaluates the performance of the dynamic AR marker. Finally, Section 6 concludes the paper and suggests future work.

## 2. Related Work

The IoT paradigm is very well suited to the architectural requirements of smart city services and has therefore been previously considered in work in this field.

Zanella *et al.* [2] designed an urban IoT network based on the web service paradigm, deployed in Padova (Italy). This proof of concept monitors street lighting and air quality and allows remote identification of issues that demand the attention of maintenance staff through either a central control station or mobile devices.

A different perspective is found in previous publications [7], where sensors and actuators of an IoT-enabled smart city scenario are considered service providers, just as in a cloud provisioning model. This proposal eliminates the barriers between physical (sensors and actuators) and logical (cloud service providers) worlds, creating a higher level of abstraction that allows the deployment of innovative ubiquitous smart city services.

Taking a framework perspective, Jin *et al.* [8] identified the basic IoT elements needed in smart cities and described a case study for noise monitoring.

Although the above works deal with the interaction through mobile devices between city staff and citizens and smart city services, they mainly focus on the communications architecture and do not describe methods to improve user interaction or proximity communications with AR technologies, as proposed in our work.

We must remark, however, that AR interfaces have already been applied in smart city and smart building scenarios to deliver context-dependent information for both maintenance tasks and other purposes, such as tourist information services [9–12]. For example, in Smart Santander, tourist information is delivered via AR interfaces on GPS-enabled smartphones, associating points of interest with cloud data. This is complemented with QRcode labels and RFID tags when a more precise location is needed [13,14].

Current smartphones allow the creation of powerful AR interfaces using video see-through display techniques [15]. To create an overlay with augmented information on top of the image shown to the user, the system needs to accurately identify and track relevant objects in the video stream field of view. This can be achieved with techniques such as video processing, RFID object labeling and user positioning.

Static AR markers have been proposed to help to visualize sensor information. Two approaches have been considered: the placement of AR markers over the sensor nodes themselves [16,17] or elsewhere, using the marker as a gate identifier to access sensor information [18,19].

In the field of dynamic markers, we are only aware of the work by Peiris *et al.* [20], who proposed an AR marker with limited information printed using temperature-sensitive ink. This thermochromic marker enabled the devising of a four-range temperature sensor. In a smart city IoT environment, this would pose scalability and connectivity problems.

Finally, while various authors have combined AR interfaces and IoT architectures in the same framework, they only proposed static AR markers as physical object IDs [21,22]. In general, although the individual technologies that form part of our proposal have all been dealt with in considerable depth in the literature, none of the studies to date have considered the combination of AR and VLC to support proximity information services relying on IoT architectures (where the AR marker acts as an additional communications channel between a smart object and the user interface). In this paper, we show how this combination of technologies improves usability and efficiency in a smart city maintenance scenario.

## 3. Dynamic AR Marker

Several options are available to link a physical IoT node to its logical representation in the network, so that end user applications may access this information directly through the AR user interface. Some frameworks use previously analyzed images as markers (also called anchors) to track the position and orientation of areas where augmented content needs to be added [23–25], while others use QR codes or even geopositioning to locate the points of interest.

The less computationally expensive mechanism is geopositioning. It is, however, also the least accurate option and is generally useless for scenarios where the object is centimeters or even meters from the user's smartphone. For short-range applications, such as interactive print, QR codes or image anchors are preferred for their high precision, yet they require greater computing power in user devices. Whatever the case, all of these approaches are static in the sense that the information coded in the anchor (the image, the information decoded from the QR image or the geographical position of a marker) cannot be changed at runtime. Most systems based on these approaches resolve this issue by using anchors only as a referrer to an online resource. In other words, a persistent connection is needed between the user devices and a backend infrastructure.

**Figure 1:**
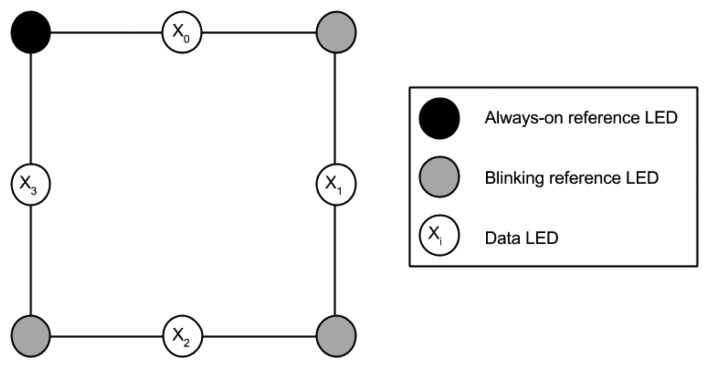
Dynamic LED marker.

In this work, we propose a new type of optical AR anchor that allows precise definition of the area of interest for media overlays while providing a direct low-bandwidth communications channel for dynamic information. To achieve this, instead of static markers based on images or QR codes, we employ an LED matrix that can be easily read by most personal device cameras nowadays. A basic diagram of this dynamic marker is shown in Figure 1.

Because of its geometric simplicity, a beacon consisting of a square array of eight LEDs was selected for a proof-of-concept implementation. The function of the LEDs in the corners of the array is to define the area to be analyzed, which is used as a reference to determine the position and orientation of the beacon. The LED in the upper left corner always remains on as a reference point to find the other three vertices and, hence, the transmitting LEDs. The remaining LEDs on the vertices (upper right, lower left and lower right) blink at a fixed frequency (*F*_1_), as a clock reference for synchronizing the sampling of the data LEDs. The four remaining LEDs, positioned halfway between the reference LEDs, are used to transmit information (data LEDs).

The four transmitting LEDs follow a pattern depending on the value to be transmitted. Each transmitting LED corresponds to a bit, in such a way that information is partitioned in four-bit chunks, equivalent to nibbles. Thus, the values sent range from 0*x*0 to 0*xF*, expressed in hexadecimal format (0*b*0000 and 0*b*1111 in binary format). Each of the four LEDs *X_i_* is assigned a weight 2*^i^*, so that the transmitted nibble *X* can be calculated simply as defined in Expression (1), where *X_i_* is zero for a dark LED and one for a lit LED.

(1) $$X=\sum_{i=0}^{3}X_{i}2^{i}$$

Care must be taken when reading the values of each *X_i_* so that the smartphone camera does not sample the status of a changing LED (ON to OFF or OFF to ON), since this could lead to errors in the decoded message. To prevent this from happening, the blinking reference LEDs are used as a clock source, so that data LEDs only change their values when the blinking reference LEDs are OFF. Furthermore, the value of each *X_i_* is only assumed to be valid if the blinking reference LEDs for the frame being processed are all ON.

**Figure 2:**
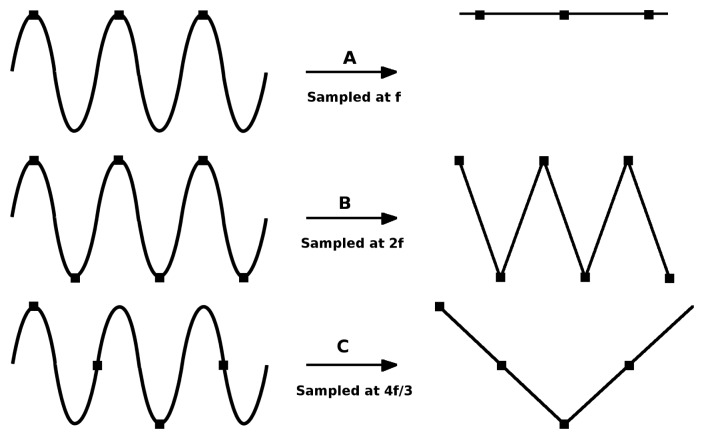
Effects of various sampling rates while sampling a signal [27].

According to this symbol coding scheme, the information transmission data rate for a given frequency *F*_1_ is *F*_1_/2 bytes per second (half a byte every 1/*F*_1_ seconds). Since most modern smartphones and video conferencing cameras are capable of shooting video at 30 frames per second [26] (*i.e.*, the sampling frequency is *F_s_* = 30*Hz*) and taking into account the sampling theorem, the maximum LED blinking frequency that can be correctly detected is *F_b_* = *F_s_*/2 = 15*Hz*. As shown in Figure 2, at least two samples (images) must be captured each blinking period in order to correctly detect the LED frequency of reference.

In order to make the system more robust against spurious external light reflections and to avoid errors, the value of each bit must be observed for three consecutive frames for the system to consider it valid. The working frequency of the data transmission system is thus *F*_1_ = *F_b_*/3 = 5 Hz. This means that, using this transmission channel, a symbol of four bits can be transmitted five times per second, which results in an overall throughput of 20 bits per second or 2.5 bytes per second. This was sufficient to validate the proposed application in a real scenario.

**Figure 3:**
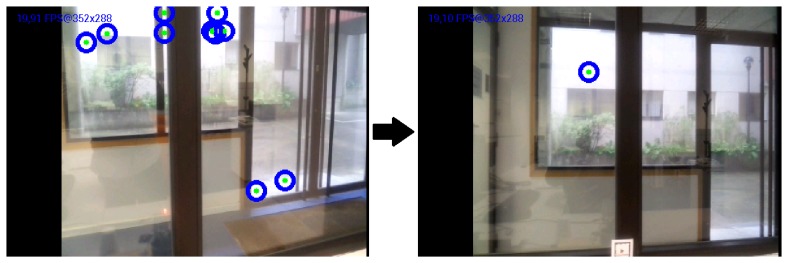
Intense light reflections may cause false positives **(Left)**. False positives are reduced by applying simple image processing techniques **(Right)**.

To enable detection of the emitted LED pattern, we implemented a simple image processing routine using OpenCV libraries [28]. First, the RGB image taken from the smartphone camera is converted to grayscale. The FindContours [29] function then locates the frame contours and stores each one as a vector of points. Once the contours of each LED have been identified, the Moments [30] function is used to return the area described by each of the stored contours. Next, the center of these areas is calculated so that each LED is referenced by a pair of *x* and *y* coordinates. However, even though LEDs are correctly located with the described method, problems may arise in highly illuminated environments. For example, light reflections or direct sunlight may give rise to false positives. To solve this problem, the detection system was improved by eliminating higher and lower contrast regions within a fixed range. Open contours were eliminated, yielding a procedure that is more robust in high luminosity conditions (Figure 3). Finally, once the LEDs have been detected and positioned, the message is decoded. The following restrictions were imposed to improve decoding reliability:

- All messages start with the byte 0*x*00.
- Message length is predefined and fixed.

When the mobile device camera is aimed at the beacon and the application starts capturing frames, these frames are analyzed until a single lit LED is found. This corresponds to the fixed reference LED located in the upper left corner of the beacon.

This LED can be detected because (1) the three LEDs located in the other corners of the beacon blink at a fixed frequency and (2) all messages start with a 0*x*00 code, with all emitting LEDs off. Thus, all other LEDs are turned off at given times. This serves as a start-of-frame (SOF) marker. Consequently, the byte 0*x*00 should not be included in the message, and therefore, a byte stuffing algorithm, such as COBS [31], is needed to escape illegal characters in the encoded sequence.

When the fixed reference LED is detected, its position is stored in memory. More captures are made until four lit LEDs (the fixed LED and the three blinking reference LEDs) are located. Next, some basic trigonometric operations are performed to verify that the four detected LEDs are arranged in a square, and the positions of the other three corner LEDs are also stored in memory.

**Figure 4:**
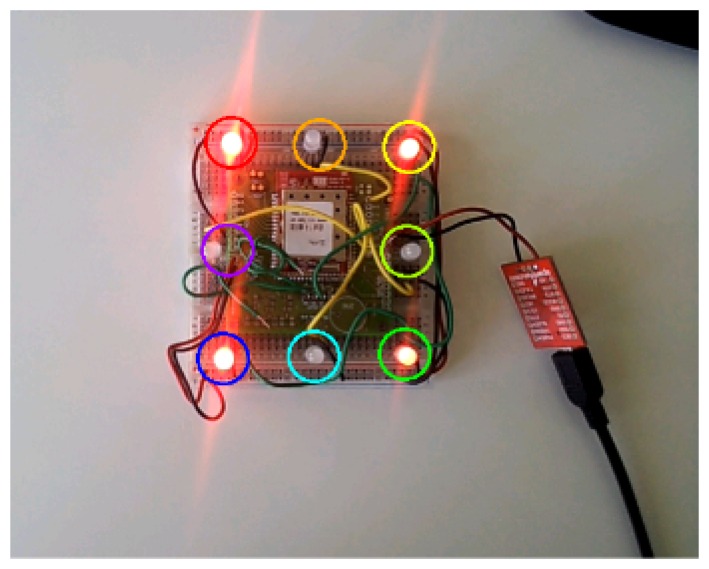
Sampling points for fixed, blinking and data LEDs.

Once the positions of the beacon corners have been identified, the middle points of the edges of the square are calculated. The locations of the data LEDs are estimated to be at these four points, as shown in Figure 4.

**Figure 5:**
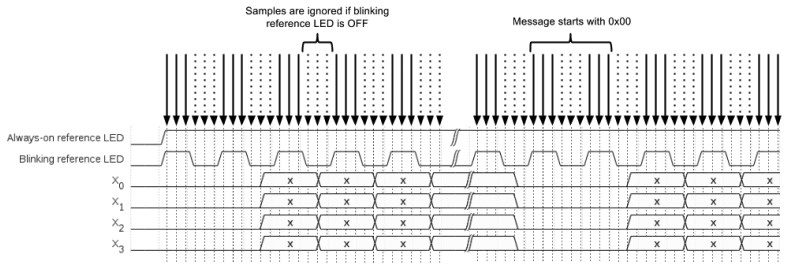
Sampling sequence for dynamic augmented reality (AR) marker LEDs.

In subsequent captures, the status of all blinking reference LEDs is checked prior to evaluating the status of the data LEDs. If the blinking reference LEDs are all lit, the values provided by the four data LEDs are stored in a vector as the transmitted symbol. Otherwise, those values are discarded in order to avoid erroneous decoding due to transient states. As previously mentioned, the system ignores symbols that are not repeated over at least three consecutive frames (Figure 5).

**Figure 6:**
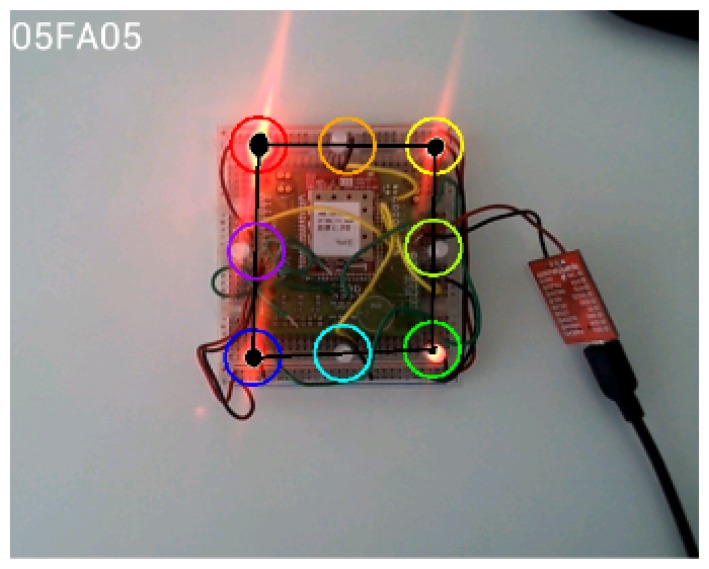
A geometrical reference is passed to the application so it can determine the relative positions of the camera and the dynamic marker.

When the number of correctly received values matches the pre-established length of the message, the message is completely decoded and passed as an input to the AR application. The positions of the corners that define the relative orientation of the marker are also sent to the application as a geometrical reference for AR overlaying tasks (Figure 6).

## 4. Architecture and Implementation

**Figure 7:**
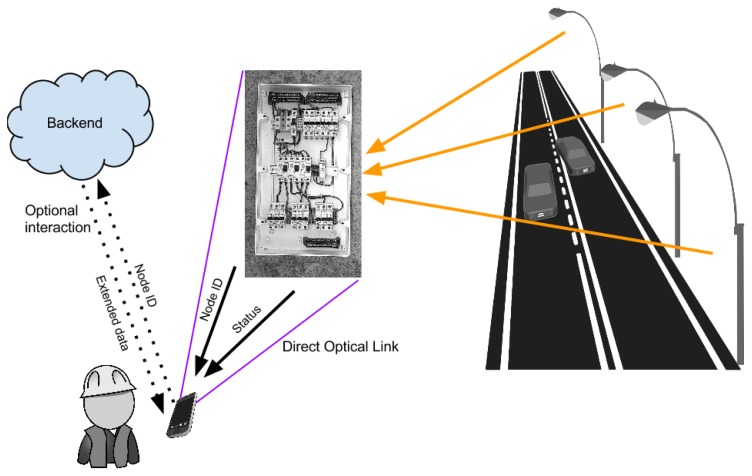
Use case for system validation.

The following use case was defined to validate the proposed technologies and their integration in a smart city scenario (Figure 7):

- A maintenance worker wishes to check the status of some city appliances.
- The worker approaches an electrical panel and points a smartphone at an AR dynamic marker.
- The smartphone decodes the information sent by the AR marker and obtains the device ID of the electrical panel and dynamic status information (average energy consumption and status codes).
- This information is shown to the maintenance worker via the AR interface.
- If additional data are needed, the smartphone application can use the device ID obtained from the AR marker to send a query to the backend infrastructure. Note that this is not compulsory in many cases, as the status codes will quite often be enough to inform the worker of the situation.

This use case represents a system that is capable of linking a physical resource (an electrical panel) to its logical representation (the panel ID) for AR applications and, at the same time, takes advantage of the same identification channel to exchange basic information without the need for a permanent WAN connection, saving thus both terminal power and bandwidth.

Thus, the system in the use case consists of a combination of three main components, namely:

- An IoT infrastructure (described in Section 4.1) capable of gathering information from several distributed monitoring points and distributing this via a low power wireless mesh communications network to several devices, where it is consumed.
- An AR user interface (described in Section 4.2), supported by the user's smartphone, that gives the user seamless access to infrastructure information.
- An AR anchor (described in Section 3) that allows a physical point of information to be dynamically linked to the logical information associated with that point.

The combination of these three elements lays the foundations for building an application that is both more intuitive for users and more efficient in terms of network resource usage thanks to a distributed access to information.

The core contribution of this paper is a novel interaction mechanism for smart city proximity information, with a use case focused on maintenance tasks, based on the combination of AR and IoT through the previously described dynamic AR anchor. However, for the system to be successfully validated in a testbed, an IoT communications infrastructure and an AR user interface are also required. Sections 4.1 and 4.2 describe these complementary elements.

### 4.1. IoT Infrastructure

The IoT infrastructure supports a population of wireless nodes deployed in a smart city environment, capable of sensing several parameters depending on the maintenance procedures. A backbone city network of any type is used for central data transport.

Furthermore, a wireless infrastructure is available to provide the smartphones of the citizens and maintenance workers with network access through several access points (WiFi APs or base stations) scattered throughout the city.

Finally, sensing nodes are placed in several infrastructure elements, such as lamp posts, traffic lights or electrical panels. These sensors collect information on parameters such as power consumption or performance metrics. When arranged in local clusters, sensing nodes form local mesh networks in order to communicate directly with each other.

**Figure 8:**
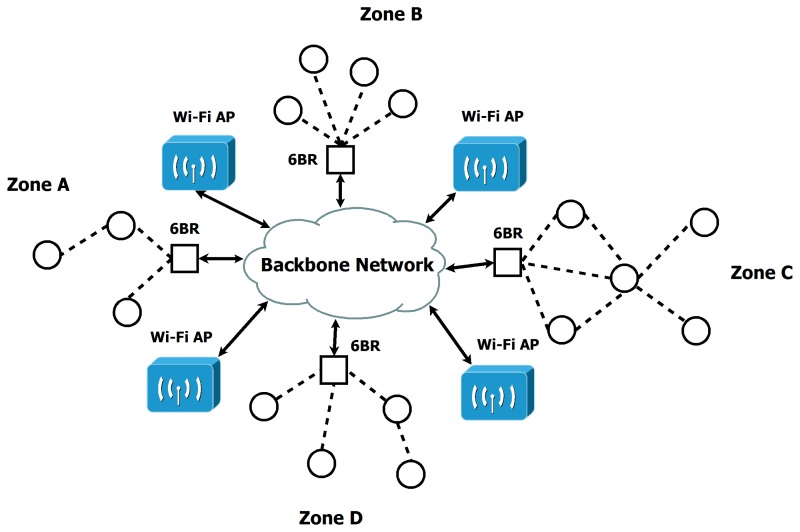
Architecture model of the IoT infrastructure.

In this scheme, at least one of the nodes in each mesh area acts as a border router (BR) to communicate the group with the wired backbone and hence with the central servers and the cloud. Figure 8 summarizes this architecture model.

Each node uses 6LoWPAN and IPv6 protocols to communicate with other devices. This choice is coherent with the fact that sensor nodes may be battery powered and have limited computation resources. Low power technologies, such as 6LoWPAN, can be applied to network data transfers while maintaining a high degree of flexibility, scalability and energy efficiency. We chose to use the Contiki operating system [32] for the nodes, as it is specially designed for constrained devices and includes many *de facto* IoT communication standards

Sensor measurements are not expected to use more than 80-byte messages, and the transmission frequency of the nodes is expected to be low, so high data rates are not necessary. We therefore used an IEEE802.15.4 [33,34] wireless radio for mesh area communications. Adjacent areas use different radio channels to avoid interference. Moreover, all areas are supposed to have their own PAN ID, so that they are mutually independent.

The network layer employs the 6LoWPAN protocol [35]. This allows each node to have its own IPv6 address and to be directly accessed from any other node or device implementing the IPv6 protocol.

The BRs located in each of the defined mesh zones are responsible for routing information from their respective mesh networks to the backbone and for ensuring adaptation between the 6LoWPAN protocol and the standard IPv6 protocol. They maintain route tables for their networks and are also in charge of 6LoWPAN header compression and decompression for messages sent through their interfaces between the 6LoWPAN network and the backbone.

The routing protocol of the 6LoWPAN network is the RPL [36] distance vector routing protocol. It builds routes to communicate with nodes within the same mesh area. RPL was chosen for its efficient memory usage and low control traffic relative to other options, such as link state routing protocols.

**Figure 9:**
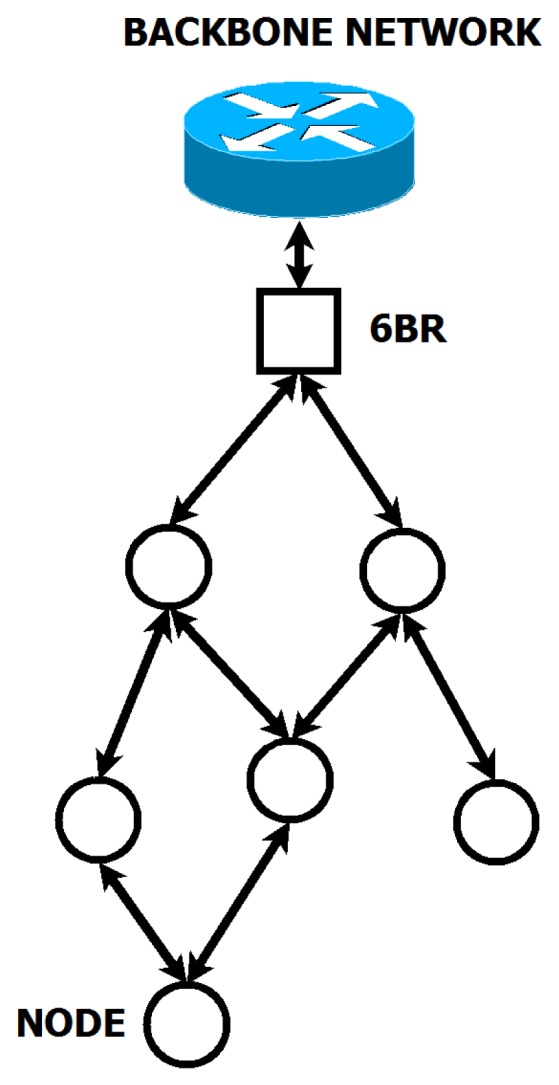
Architecture model of the IoT infrastructure. Detail of mesh area network.

In Figure 9, the border router acts as the root node of the destination-oriented directed acyclic graph (DODAG) built by the routing protocol. The objective function used to build the DODAG is the minimum rank with hysteresis objective function (MRHOF), which uses the estimated transmission count (ETX) as the default metric. This metric estimates the number of transmissions until a message reaches its destination and builds the graph accordingly. When possible, redundant paths are established to avoid node isolation.

**Figure 10:**
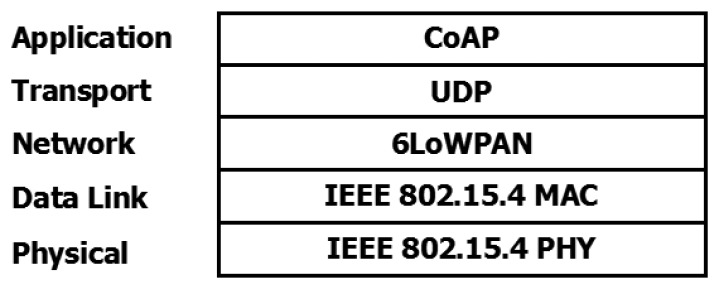
6LowPAN protocol stack.

The CoAP protocol [37] was chosen for the application layer, using UDP as the transport protocol (Figure 10). This protocol allows the implementation of REST interfaces over constrained networks formed by nodes with limited resources. Thus, each node can be accessed and its resources modified using CRUD (create, read, update and delete) methods. Moreover, CoAP has been designed to support HTTP bridging, so external devices (outside the network) not implementing the CoAP protocol may access node resources directly if an HTTP/CoAP bridge is included in the network.

### 4.2. AR User Interface

We designed an AR graphic user interface (GUI) to exemplify the possibility of good user experience with the system. The GUI is used to read and update information in the cloud infrastructure.

One of the main advantages of using AR along with a dynamic anchor is the possibility of visualizing real-time data by simply pointing the user terminal to the target object (node), even when a connection with the backbone network is not available. For that purpose, the terminal receives the following data through an optical channel:

- ID number of the node.
- Status code of the node.
- Average power consumption over the last measuring period.

All of this information can be displayed as soon as the node is detected and the VLC message is decoded. The transmitted message is composed of a three-byte node ID, followed by an eight-bit status code (0*x*00 meaning OK) and a 16-bit integer value representing average power consumption (Table 1).

**Figure 11:**
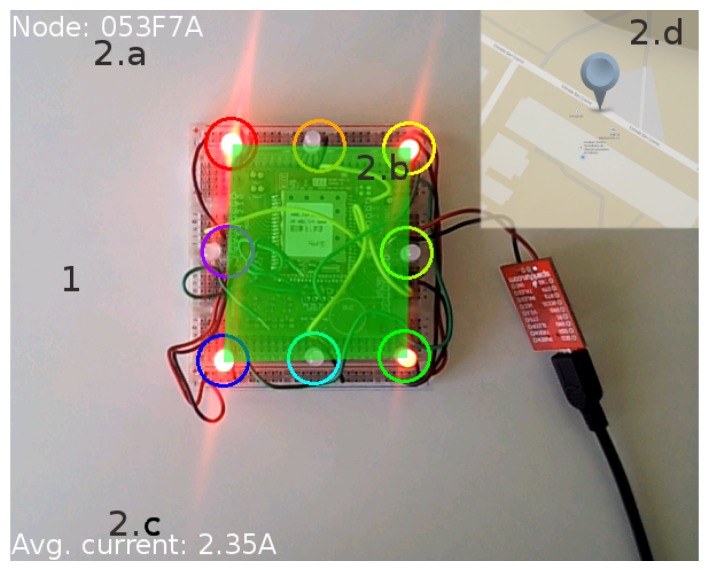
Information presented to the user via the AR GUI.

As shown in Figure 11, the information is presented to the user as follows:

1. GUI background
  (a) The actual image captured by the device camera is shown as the GUI background.
2. AR superimposed information.
  (a) Node ID: The top-left corner of the screen shows the unique identification number of the detected node. This allows interaction with the node through the cloud.
  (b) Node status: This represents the current status of the target node. A semi-transparent color-coded rectangle is used, spread between the four corners of the dynamic anchor. It also acts as a button to get extended information. The color of the square is related to node status:
    i. Green: All of the electrical features of the node are correct.
    ii. Yellow: A problem has not yet been detected, but the readings exceed the normal ranges. A maintenance worker should check the node to prevent further damage.
    iii. Red: The node has a problem with one or more of its connections. A maintenance worker should repair the panel.
  (c) Average power: The bottom-left corner displays the average power consumption measured over the last measurement period.
  (d) Location map: A semi-transparent map is displayed in the top-right corner of the screen showing the maintenance worker the location of the problem. This information is recovered from the cloud services if available. Otherwise, the map is not shown.

**Figure 12:**
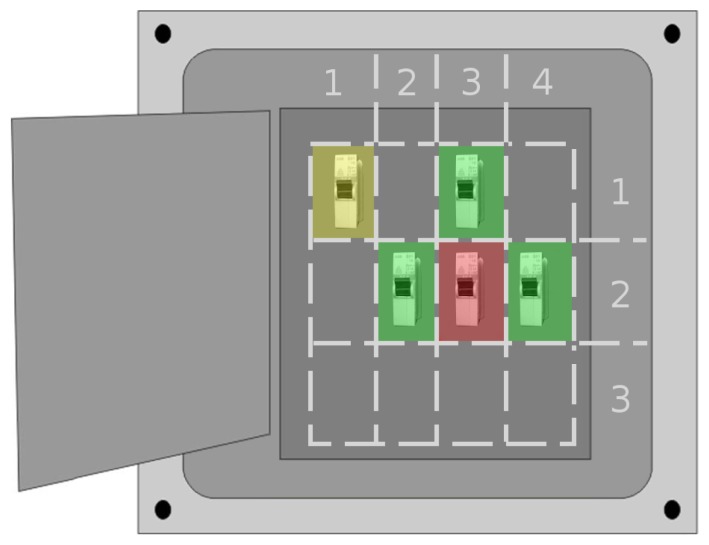
Design for a more detailed user interface.

This general GUI provides a quick overview of what is happening inside the target node. Nevertheless, the proposed system has much more potential, which can be exploited with a GUI mode with finer detail. In order to access this detailed information, an extended interface is proposed that would be displayed by touching the colored zone over the dynamic anchor image. Figure 12 shows an example for the design of this finer GUI.

When the maintenance worker changes the view from general to detailed information, the incoming information is fed to the terminal in a different way. Whilst general data are mainly received through optical (VLC) means, detailed information is retrieved by querying the cloud with the corresponding node ID.

In our example, each electrical panel has several wired connections, and the GUI shows their individual power consumption, status and the procedure for closing or opening the connection. To this end, the relative position of the node with respect to the dynamic anchor is used as a reference to form a grid. Some of the panel's cells will contain a connection, easily identified by the grid coordinates, and can be selected to obtain more detailed information.

By dividing the electrical panel image in cells, it is easy to show which connections are functioning properly and which need to be repaired. For instance, a semitransparent colored region can be superimposed over each connection. The color code can follow the same pattern as the general GUI, applied at the connection rather than the overall node level. For example, in Figure 12, there are connections in (1,1), (3,1), (2,2), (3,2) and (4,2), but only the connections in (1,1) and (3,2) exhibit anomalies.

**Figure 13:**
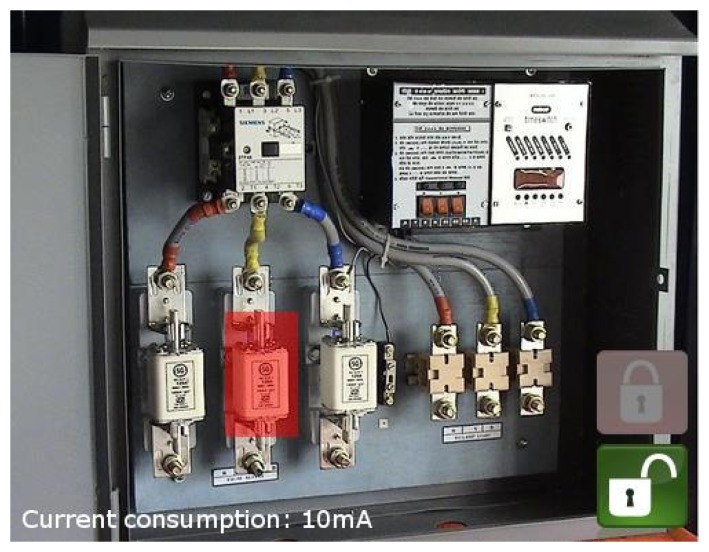
Example of a detailed view with available connections and related controls.

In addition to providing an adequate reflection of individual connection status, the grid division also allows the creation of buttons to request information on and interact with a given connection. When the maintenance worker clicks on one of the cells with a connection, the most detailed view will be displayed (Figure 13). This interface offers the following options:

- Status of the target connection: only the cell of the selected connection will be enabled following the same color code as in the other modes of the GUI.
- Power consumption of the connection: the bottom-left corner of the screen shows the power consumption of the connection in real time.
- The bottom-right corner of the interface displays two buttons to open and close the target connection. If the connection is closed, the “close” button will be disabled (as in Figure 13), and if it is open, the “open” button will be disabled. Clicking the enabled button will switch the connection on or off. This is the only option in which the terminal can change the state of the node.

## 5. Validation and Results

**Figure 14:**
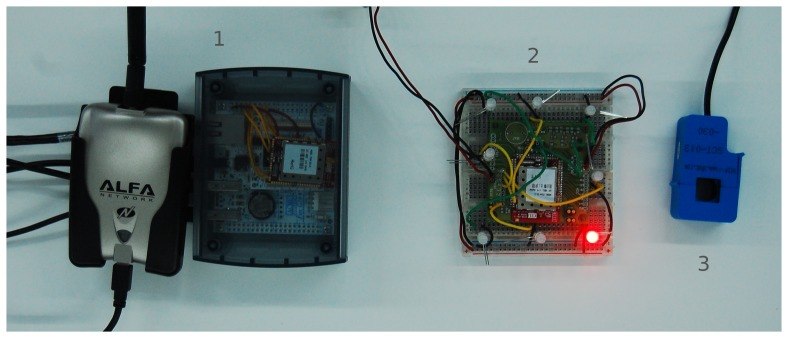
Main components of the laboratory testbed: (1) border router and gateway; (2) dynamic marker; (3) amperometric clamp.

We deployed a testbed to validate the systems described in this paper, emulating a real smart city infrastructure. Figure 14 shows the main components of the testbed, which are described in Section 5.1. In Section 5.2, we present the results of the performance evaluation of the dynamic AR marker.

### 5.1. Testbed Components Identification

The testbed was built over an Ethernet LAN emulating the wired backbone network with a WiFi access point, allowing access to external terminals, such as smartphones. A 6LoWPAN border router was connected to the network to route traffic from several test nodes and beacon prototypes.

The radio module used in the testbed nodes was the Freestar Pro ZFSM-201-X [38] from California Eastern Laboratories. It includes a microcontroller unit (MCU), a transceiver and an antenna, integrated in a printed circuit board. It is based on Freescale's MC13226V [39] platform and incorporates a power amplifier (PA), a printed antenna and a connection interface for prototyping in other boards.

This module was included in a board designed to easily connect a variety of sensors and actuators to the MCU ADC. Moreover, several LEDs were connected to that board in order to implement the dynamic marker beacon described in Section 3.

Each node in the IoT network ran a version of the Contiki OS, ported to MC13226V microcontrollers, with the uIP [40] protocol stack and CoAP as the application layer protocol. We have implemented the following resources with CoAP for the nodes in the testbed, which are to be placed in electrical panels:

- *well-known/core:* default resource used by CoAP to request the list of resources for a node;
- *sensors/vbatt:* to monitor battery level;
- *sensors/connstatus:* to indicate the status of the indicated electrical connection of the panel;
- *sensors/energycons:* to obtain the average power consumption through the selected electrical connection;
- *actuators/set_beacon_freq:* to set the frequency of the transmitting LEDs;
- *actuators/led_pos:* to activate the reference LEDs in the corners;
- *actuators/led_beacon:* to activate the data transmission LEDs;
- *options/rfchannel:* to modify the IEEE802.15.4 radio channel;
- *options/txpower:* to modify the transmission power of the node;

These resources are directly accessible on each node via the WAN interface of the border router. Accordingly, any device connected to the backbone network can access relevant real-time information provided by any of the individual infrastructure elements.

**Figure 15:**
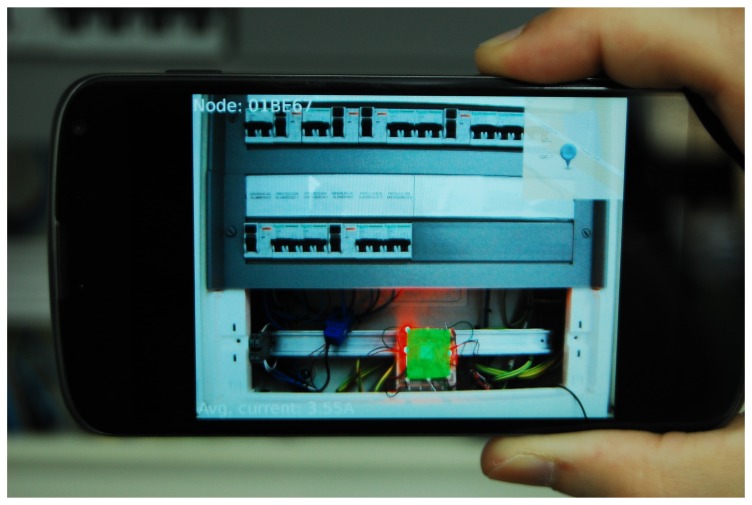
AR application in the testbed deployment showing the status of an electrical panel.

In the next step, we connected the test nodes to the electrical panels of a building using amperometric clamps to measure the total intensity of current flow. These nodes were equipped with prototypes of dynamic AR markers, allowing us to deliver basic information about the connections to an Android AR application (Figure 15).

For the border router prototype, the following modules were used:

- ZFSM-201-2 California Eastern Labs MCU with Freescale MC123226V microcontroller
- FOX Board G20 [41] with Debian Linux system

The same radio module was used in the test nodes and the 6LoWPAN border router. Moreover, to act as a bridge between wired (Ethernet) and wireless networks (WiFi), this module was connected to a Linux board for routing traffic from these interfaces according to the destination subnet. This was calculated using the IPv6 address of the destination (the same applied when routing traffic in the opposite direction). Thus, interconnections between all of the elements of the testbed are supported, thereby facilitating the inclusion of new nodes in the future.

Finally, the AR GUI in Figure 15 was implemented for Android devices. In order to interact with the CoAP resources in IoT nodes, the Californium framework libraries [42] were ported to Android, after resolving several problems regarding inter-process communications and library compatibility.

Using these ported libraries together with the OpenGL ESAPI [43], we implemented and tested the basic information GUI described in Section 4.2 in a Nexus 4 terminal. Although the finer extended GUIs described in Section 4.2 were left for future implementation, this prototype allowed us to validate direct VLC communication between the user terminal and a node, as well as cloud information retrieval.

### 5.2. Dynamic AR Marker Performance

**Figure 16:**
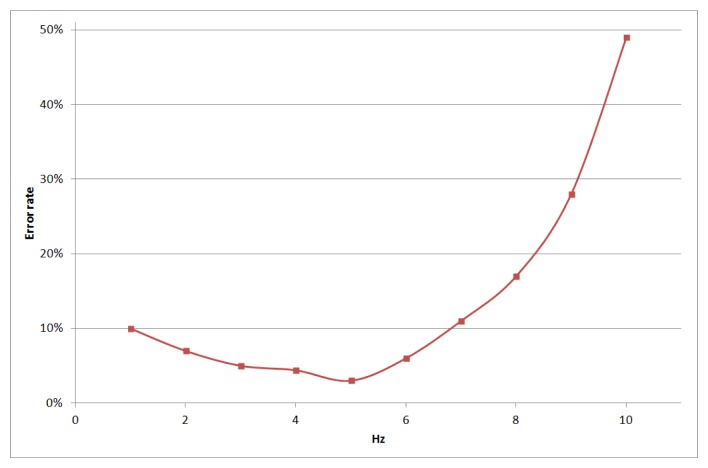
Error rate of the VLC message *versus* marker blinking frequency.

The first tests sought to find the optimum blinking frequency of the dynamic AR marker, to maximize data throughput for an acceptable error rate. This frequency was estimated to lie in the interval between 3 and 6 Hz, as shown in Figure 16. Inside this range, the best frequency was ∼5 Hz.

**Figure 17:**
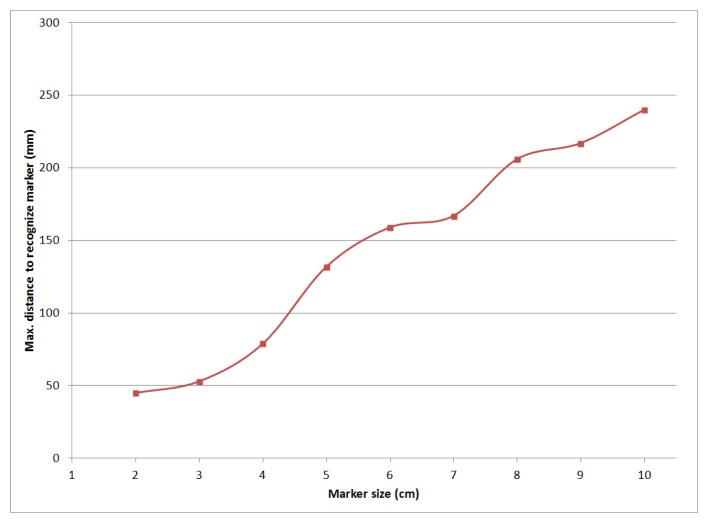
Maximum dynamic marker detection distance *versus* marker size.

Another relevant performance evaluation target was the maximum detection distance, to assess the usability of the system proposed against other technologies that cannot attain both high directionality and relatively long ranges, such as NFC, RFID, WiFi and Bluetooth. This distance is directly proportional to the actual size of the marker. Figure 17 shows the maximum detection distances for our mobile device, for dynamic marker sizes between 2 cm and 10 cm. Smaller sizes would be unfeasible, as the resulting distances would not be valid for AR applications. Nevertheless, bigger sizes would be indeed feasible and even desirable, if longer detection distances are necessary. For example, it would be possible to arrange the marker LEDs around the electrical panel. The prototype presented in this paper used a 6 × 6 cm marker yielding a maximum interaction distance of about 160 cm.

**Figure 18:**
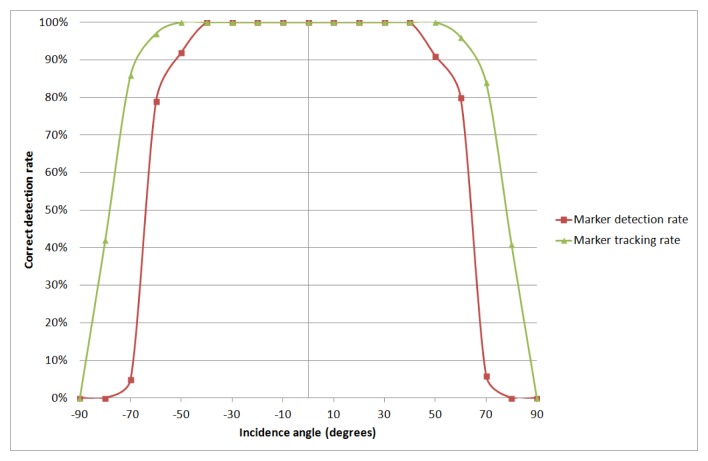
Dynamic marker detection rate *versus* camera incidence angle.

We also evaluated the effect of the device camera incidence angle in the detection rate of the dynamic marker. We tested this both for detection and tracking. Figure 18 shows the results. They suggest robust detection is feasible for angles between −50 and 50 degrees, which is perfectly adequate for the purposes of the system. By adding tracking to the recognition, this range widened to (−60, 60).

The testbed demonstrates the interest and feasibility of the combination of IoT and AR technologies and highlights the advantages in terms of usability and information availability that can be achieved with our proposal.

## 6. Conclusions and Future Work

In this paper, we have proposed and described a novel approach to link real-world objects (with sensor and actuator capabilities) to virtual world information relying on proximity communications. The system combines dynamic AR markers (which can also be used to locate objects and estimate their spatial orientation in video streams), VLC channels and a supporting IoT architecture. VLC has been chosen *versus* other communication technologies, such as RFID/NFC, Bluetooth and WiFi, due to its more extended hardware support, better accuracy for AR positioning and relatively long range. These components allow one to represent the sensors and actuators in a smart city as services that can be easily accessed via graphical interfaces based on real-world images, without the need for permanent connection with the backbone network. As described, this new approach may help to improve maintenance tasks in smart cities. Furthermore, our architecture can be easily adapted to other use cases. For example, providing AR information to tourists, obtained from different sources of urban information, or providing technical AR information to workers in industrial environments.

Future extensions of this work will consider mechanisms to reduce the decoding latency of marker VLC messages, implement the finer AR user interfaces we have described, introduce new node actions and apply our technologies to other interesting use cases.

## Figures and Tables

**Table 1:** Visible light communication (VLC) message fields. SOF, start-of-frame.

**SOF**	**Byte 0**	**Byte 1**	**Byte 2**	**Byte 3**	**Byte 4**	**Byte 5**
0x00		Node ID		Status code	Average	energy

## References

[1] Schaffers H., Komninos N., Pallot M., Trousse B., Nilsson M., Oliveira A. (2011). Smart cities and the future internet: Towards cooperation frameworks for open innovation. The Future Internet, Lecture Notes in Computer Science.

[2] Zanella A., Bui N., Castellani A.P., Vangelista L., Zorzi M. (2014). Internet of Things for Smart Cities. IEEE Internet Things J..

[3] Santander on FIRE Future Internet Research and Experimentation. http://www.smartsantander.eu/.

[4] Doran M.A., Daniel S. (2014). Geomatics and Smart City: A transversal contribution to the Smart City development. Inf. Polity.

[5] Gil-Castiñeira F., Costa-Montenegro E., González-Castaño F.J., López-Bravo C., Ojala T., Bose R. (2011). Experiences inside the Ubiquitous Oulu Smart City. Computer.

[6] Weiser M. (1991). The computer for the 21st century. Sci. Am..

[7] Mitton N., Papavassiliou S., Puliafito A., Trivedi K.S. (2012). Combining Cloud and sensors in a smart city environment. EURASIP J. Wirel. Commun. Netw..

[8] Jin J., Gubbi J., Marusic S., Palaniswami M. (2014). An information framework for creating a smart city through Internet of things. IEEE Internet Things J..

[9] Museum of London. Street Museum http://www.museumoflondon.org.uk/Resources/app/you-are-here-app/noflash/no-flash.html.

[10] Mason J. Something in the AIR in Madison Square: Smarter Cities and Augmented Reality. http://asmarterplanet.com/blog/2010/05/something-in-the-air-in-madison-square-smarter-cities-and-augmented-reality.html.

[11] Romualdo S.L., Brown K., Pipes S., Ibbotson J. Smart building management through augmented reality.

[12] Koch C., Neges M., Konig M., Abramovici M. (2014). Natural markers for augmented reality-based indoor navigation and facility maintenance. Autom. Construct..

[13] Jose A.G., Gutiérrez V., Santana J.R., Sánchez L., Sotres P., Casanueva J., Muñoz L. SmartSantander: A Joint Service Provision Facility and Experimentation-Oriented Testbed, within a Smart City Environment 2013. http://www.smartsantander.eu/downloads/Presentations/SmartSantander_A_joint.pdf.

[14] Sánchez L., Gutiérrez V., Galache J., Sotres P., Santana J., Casanueva J., Muñoz L. SmartSantander: Experimentation and service provision in the smart city.

[15] Van Krevelen D., Poelman R. (2010). A survey of augmented reality technologies, applications and limitations. Int. J. Virtual Real..

[16] Wada K., Kawahara Y., Asami T. A design of XML schema for information presentation system using augmented reality in new generation network management.

[17] Sato K., Sakamoto N., Shimada H. (2015). Visualization and Management Platform with Augmented Reality for Wireless Sensor Networks. Wirel. Sens. Netw..

[18] Goldsmith D., Liarokapis F., Malone G., Kemp J. Augmented Reality Environmental Monitoring Using Wireless Sensor Networks.

[19] González F.C.J., Villegas O.O.V., Ramírez D.E.T., Sánchez V.G.C., Domínguez H.O. (2014). Smart Multi-Level Tool for Remote Patient Monitoring Based on a Wireless Sensor Network and Mobile Augmented Reality. Sensors.

[20] Peiris R.L., Fernando O.N.N., Cheok A.D. A Dynamic AR Marker for a Paper Based Temperature Sensor.

[21] Barthel R., Hudson-Smith A., de Jode M., Blundell B. Tales of Things The Internet of “Old” Things: Collecting Stories of Objects, Places and Spaces 2010.

[22] Pokric B., Krco S., Pokric M. Augmented Reality Based Smart City Services Using Secure IoT Infrastructure.

[23] Layar. Augmented Reality | Interactive Print https://www.layar.com/.

[24] Qualcomm Connected Experiences Inc. Vuforia Developer Portal https://developer.vuforia.com/.

[25] Metaio. Junaio Augmented Reality Browser http://www.junaio.com/.

[26] Pang G.K., Liu H.H. (2001). LED location beacon system based on processing of digital images. IEEE Trans. Intell. Transp. Syst..

[27] National Instruments Analog Sampling Basics. http://www.ni.com/white-paper/3016/en/.

[28] Bradski G., Kaebler A. (2008). Learning OpenCV: Computer Vision with the OpenCV Library.

[29] OpenCV dev Team Structural Analysis and Shape Descriptors: Findcontours. http://docs.opencv.org/modules/imgproc/doc/structural_analysis_and_shape_descriptors.html?highlight=findcontoursfindcontours.

[30] OpenCV dev Team Structural Analysis and Shape Descriptors: Moments. http://docs.opencv.org/modules/imgproc/doc/structural_analysis_and_shape_descriptors.html?highlight=momentsmoments.

[31] Cheshire S., Baker M. (1999). Consistent overhead byte stuffing. IEEE/ACM Trans. Netw. TON..

[32] Dunkels A., Schmidt O., Finne N., Eriksson J., Österlind F., Tsiftes N., Durvy M. The Contiki OS: The Operating System for the Internet of Things, 2011. http://www.contiki-os.org/.

[33] IEEE (2012). IEEE Standard for Local and Metropolitan Area Networks Part 15.4: Low Rate Wireless Personal Area Networks (LR-WPANs) Amendment 2: Active Radio Frequency Identification (RFID) System Physical Layer (PHY).

[34] IEEE (2012). IEEE Draft Standard for Local and Metropolitan Area Networks Part 15.4: Low Rate Wireless Personal Area Networks (LR-WPANs) Amendment 1: MAC Sub-Layer.

[35] Shelby Z., Bormann C. (2011). 6LoWPAN: The Wireless Embedded Internet.

[36] Winter T. RPL: IPv6 Routing Protocol for Low-Power and Lossy Networks 2012 (RFC 6550). https://tools.ietf.org/html/rfc6550.

[37] Shelby Z., Hartke K., Bormann C. The Constrained Application Protocol (CoAP) 2014 (RFC 7252). https://tools.ietf.org/html/rfc7252.

[38] California Eastern Laboratories, Freestar Pro Series Transceiver Modules http://www.cel.com/pdf/datasheets/zfsm_201_l_ds.pdf.

[39] Freescale Semiconductor http://www.freescale.com/webapp/sps/site/prod_summary.jsp?code=MC13226V.

[40] Dunkels A. The uIP Embedded TCP/IP Stack: 2006. http://citeseerx.ist.psu.edu/viewdoc/download?rep=rep1type=pdfdoi=10.1.1.154.2510.

[41] ACME Systems FOX Board G20—Linux Embedded SBC. http://www.acmesystems.it/FOXG20.

[42] Kovatsch M., Research Group for Distributed Systems, Swiss Federal Institute of Technology Zurich, Californium (Cf) CoAP framework in Java http://people.inf.ethz.ch/mkovatsc/californium.php.

[43] Khronos Group OpenGL ES: The Standard for Embedded Accelerated 3D Graphics. https://www.khronos.org/opengles/.

